# Exposure of pre-school aged children to schistosomiasis: a call for public health strategies

**DOI:** 10.1093/eurpub/ckac131.398

**Published:** 2022-10-25

**Authors:** J Hameister, MJ Solonirina, T Rasamoelina, R Rakotozandrindrainy, R Rakotoarivelo, P Rausche, DI Puradiredja, J May, D Fusco, freeBILy consortium

**Affiliations:** 1 Department of Infectious Diseases Epidemiology, Bernhard Nocht Institute for Tropical Medicine, Hamburg, Germany; 2 German Center for Infection Research, Hamburg-Borstel-Lübeck-Riems, Germany; 3 Department of Microbiology and Parasitology, University of Antananarivo, Antananarivo, Madagascar; 4 Charles Mérieux Center of Infectious Disease, University of Antananarivo, Antananarivo, Madagascar; 5 Department of Infectious Diseases, University of Fianarantsoa Andrainjato, Fianarantsoa, Madagascar

## Abstract

**Background:**

Schistosomiasis is a disease of poverty, prevalent in tropical regions and emerging in Europe. Mass drug administration (MDA) is a common strategy to fight the disease. Pre-school aged children (PSAC) are conventionally considered not to be at high risk of infection and thus usually excluded from MDA. However, chronic infections at young ages can result in serious health consequences. Our study aims to better understand the schistosomiasis transmission routes in PSAC and risk perceptions among caretakers in the endemic context of Madagascar.

**Methods:**

This is a cross-sectional sub-study embedded in the clinical trial freeBILy. Using a mixed methods approach, 86 women and their two-year-old children in four communities of the Amoron’ I Mania and Itasy regions of Madagascar in April 2022 were interviewed using structured questionnaires, focus group discussions and in-depth-interviews. Additionally, structured observational data were collected on the behaviours of 20 PSAC around waterbodies in the communities where notes and anonymised photos were taken.

**Results:**

Preliminary analysis shows that knowledge of schistosomiasis, its transmission routes and effects is limited. Risky behaviours of children were reported during the observations. They joined their mothers during daily activities and spent between 3-60 mins in rivers, lakes and rice fields. The exposure time to potentially contaminated waters was sufficient to represent a risk for infection.

**Conclusions:**

Even though our preliminary results show that PSAC are at risk of schistosomiasis infection, they are currently excluded from systematic public health interventions. The lack of risk awareness among caretakers in endemic countries calls for actions to establish tailored programs on health literacy and education. Treatment should be considered for PSAC as well as the implementation of awareness campaigns, both locally and globally given the re-occurrence of schistosomiasis in countries outside the tropics.

**Key messages:**

• Pre-school aged children are exposed to the risk of early infection with schistosomiasis.

• Public health programs against schistosomiasis should address pre-school aged children in endemic countries and in health promotion strategies globally.

